# Limiting Factors in Treatment Success of Biofilm-Forming Streptococci in the Case of Canine Infective Endocarditis Caused by *Streptococcus canis*

**DOI:** 10.3390/vetsci10050314

**Published:** 2023-04-25

**Authors:** Miriam Katsburg, Christiane Weingart, Etienne Aubry, Olivia Kershaw, Judith Kikhney, Laura Kursawe, Antina Lübke-Becker, Annette Moter, Marianne Skrodzki, Barbara Kohn, Marcus Fulde

**Affiliations:** 1 Institute for Microbiology and Epizootics, Freie Universität Berlin, Robert-von-Ostertagstr. 7, 14163 Berlin, Germany; 2 Veterinary Centre for Resistance Research (TZR), Freie Universität Berlin, 14163 Berlin, Germany; 3 Small Animal Clinic, Freie Universität Berlin, Oertzenweg 19b, 14163 Berlin, Germany; 4 Institute of Veterinary Pathology, Freie Universität Berlin, Robert-von-Ostertagstr. 15, 14163 Berlin, Germany; 5 Biofilmcenter, Department for Microbiology, Infectious Diseases and Immunology, Charité-Universitätsmedizin Berlin, Hindenburgdamm 30, 12203 Berlin, Germany; 6 MoKi Analytics GmbH, Marienplatz 9, 12207 Berlin, Germany; 7 Moter Diagnostics, Marienplatz 9, 12207 Berlin, Germany

**Keywords:** biofilm, blood culture, echocardiography, FISH imaging, bacterial characterization

## Abstract

**Simple Summary:**

Endocarditis in dogs is a rare but severe disease with a bad prognosis. Early diagnosis and improved treatment plans could be beneficial to the outcome of the treatment. However, diagnosis is difficult because of the nondiscriminatory symptoms of the disease. In this report, we describe a case of endocarditis in an 8-year-old male dog caused by *Streptococcus canis*, a bacterium that is part of the normal microbiota of a dog’s skin and mucous membranes. The treatment that he received was unfortunately unsuccessful. We decided to investigate the infected heart valve and the bacterium to understand his disease better. We discovered a biofilm on the heart valve, which explains the difficulty of treating the infection, because bacteria in biofilms are more resistant to antibiotics. We propose more research towards better treatment of bacteria in biofilms, especially in the case of endocarditis, which is such a lethal disease without a prompt and effective treatment.

**Abstract:**

An 8-year-old male Rhodesian Ridgeback was presented with fever and severe thrombocytopenia. Clinical and laboratory examination, echocardiography, blood culture, and pathohistology revealed evidence of infective endocarditis, ischemic renal infarcts, and septic encephalitis. Treatment was started immediately but the dog’s condition worsened, and the dog had to be euthanized. The causative *Streptococcus canis* strain was detected by blood culture and MALDI-TOF MS and analyzed using whole-genome sequencing and multilocus sequence typing. Antibiotic susceptibility testing did not detect any resistance. The affected heart valve was analyzed using FISH imaging, which showed a streptococcal biofilm on the heart valve. Bacteria in biofilms are recalcitrant to antibiotic treatment. Early diagnosis could be beneficial to treatment outcome. Treatment of endocarditis could be improved by researching the optimal dosage of antibiotics in conjunction with the use of biofilm-active drugs.

## 1. Case Presentation

**Figure 1:**
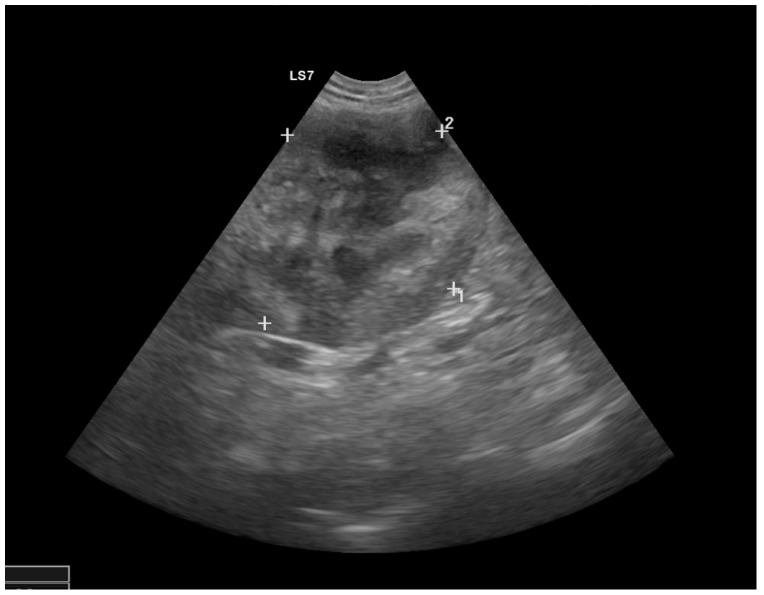
Ultrasound image of the right kidney with marked heterogeneous structural changes at the cranial pole and a hyperechoic retroperitoneal area.

An 8-year-old neutered male Rhodesian Ridgeback (weight 41.5 kg) with fever and severe thrombocytopenia had been treated with an immunosuppressive dosage of prednisolone and doxycycline for nine days due to a suspicion of immune-mediated thrombocytopenia (testing for platelet-bound antibodies was negative). Primary immune-mediated thrombocytopenia was suspected because no underlying diseases or triggering factors (e.g., neoplasia or vector-borne infections such as *Anaplasma phagocytophilum* or *Babesia canis*) were obvious at the initial evaluation. Treatment with doxycycline was started to treat possible vector-borne infections (e.g., with *Anaplasma phagocytophilum*) while the PCR result was pending. Because the dog had not traveled outside Germany, an *Ehrlichia canis* or *Leishmania* spp. infection was very unlikely. PCR testing for *Bartonella* spp. was not performed. The dog received regular ecto- and endoparasite prophylaxis, and was regularly vaccinated against canine distemper, infectious canine hepatitis, parvovirus infection, leptospirosis, and rabies. The reasons for presentation and reevaluation at the internal medicine unit of the University of Berlin were recurrence of fever, lethargy, inappetence, and lack of improvement of the thrombocytopenia. Clinical examination revealed a rectal temperature of 40.2 °C. The dog had pale pink, moderately moist mucous membranes, and a capillary refill time of 1 s. The pulse rate was 100 beats/min. The pulse was symmetrically strong and regular. The auscultation of the heart revealed regular heart sounds. A left apical pansystolic grade 3/6 murmur was auscultated. The arterial systolic blood pressure was measured at the tail using the Doppler technique (Doppler Eickemeyer, Tuttlingen, Germany). It was 120 mmHg in the quiet dog. Abdominal palpation appeared painful. On the margin of the right pinna, a small wound covered with a crust was detected. Hematological findings (Sysmex XT-2000i, Norderstedt, Germany) included severe thrombocytopenia (confirmed by manual counting), severe neutrophilia with a left shift, and monocytosis. Activated thromboplastin time (PTT) was prolonged, and prothrombin time (PT) was at the upper range of normal (Schnitger and Gross, Amelung, Lemgo, Germany). Blood chemistry (Konelab Prime 60i, Thermo Scientific, Berlin, Germany) revealed moderate azotemia; hyperphosphatemia; hyperbilirubinemia; hyperglobulinemia (55 g/L); and increased serum lipase, CRP, and cardiac troponin I concentrations (Table 1). At admission, a blood sample was taken for a blood culture (Oxoid Signal^®^, Thermo Fisher Scientific GmbH, Karlsruhe, Germany). The chest radiograph (DigitalDiagnost, Philips, Hamburg, Germany) was unremarkable. The abdominal radiograph revealed splenomegaly and a mild loss of detail in the retroperitoneal space. Ultrasonography of the abdomen (Logic S7, Scil animal care GmbH, Viernheim, Germany) revealed a heterogeneous complex mass (5 × 5 cm) at the cranial pole of the right kidney with a small amount of free fluid in the retroperitoneal space (Figure 1), a 0.4 × 0.4 cm hypoechoic lesion in the spleen without blood flow, and a painful and swollen pancreas. An abscess, abscessing neoplasm, or an infarct of the right kidney; a splenic infarct; and pancreatitis were suspected.

**Figure 2:**
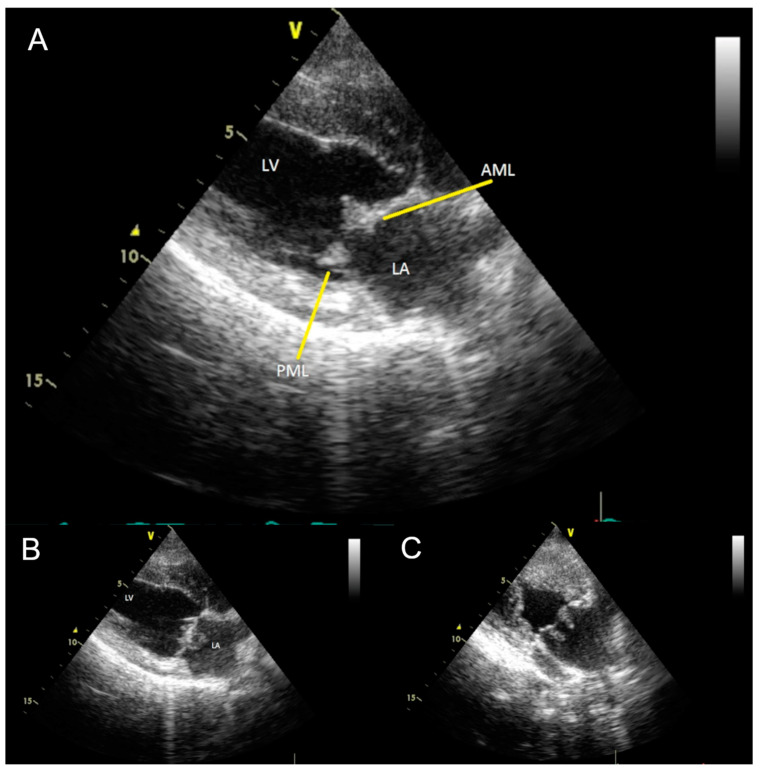
(**A**) Transthoracic two-dimensional echocardiogram in right parasternal long axis view, diastole, showing vegetations (yellow arrows) on the posterior (PML) and anterior (AML) mitral valve leaflets. LV: Left ventricle; LA: Left atrium. (**B**) Transthoracic two-dimensional echocardiogram with modified right parasternal four-chamber view to better visualize the mitral valve. (**C**) Vegetations appear as elongated hyperechoic structures on the surface of the mitral valve facing the left atrium. During echocardiographic examination, mobility of masses in the left atrium was evident.

The patient’s echocardiographic examination (Vivid 7 Dimension, Scil animal care company GmbH, Viernheim, Germany) showed mitral valve vegetation, visible as hyperechoic overlays of the unevenly thickened mitral valve (Figure 2A). The mitral valve changes were evident in diastole and in systole in the left ventricle as well as in the left atrium (Figure 2B,C). The tricuspid valve presented as mildly thickened with no discernible plaques. Both semilunar valves were unremarkable on the echocardiogram. In M-mode, all wall thicknesses and the size of the left and right ventricles as well as the right atrium were within the biological variance. The left atrium was mildly dilated. Contractility was not impaired, and the myocardium was unremarkable. Doppler sonography and color Doppler showed a severe regurgitation jet over the massively altered mitral valve. With a velocity of up to 5.6 m/s, the regurgitation jet extended to the roof of the left atrium, which it almost completely filled. The tricuspidal regurgitation jet was visible close to the leaflets’ commissure. The aortic (1.4 m/s) and pulmonary (1.15 m/s) maximal blood flow velocities, respectively, were normal. With registration over five minutes, the electrocardiogram (PC-EKG 2000, Eickemeyer, Tuttlingen, Germany) showed a sinus rhythm with a heart rate at the upper reference limit (150 beats/min) and only one left ventricular premature beat with a right bundle branch block morphology. There was an S-T segment depression in leads I and II of 0.2 to 0.3 mV.

Treatment included fluid therapy with crystalloids (Sterofundin^®^, Braun, Melsungen, Germany) (calculated for maintenance requirement 5% dehydration), metamizole (Novacen^®^ CP Pharma, Burgdorf, Germany) (10 mg/kg IV every 8 h), omeprazole (Omeprazol-ratiopharm^®^, Ratiopharm GmbH, Ulm, Germany) (1 mg/kg IV every 12 h), maropitant (Emex^®^, CP Pharma, Burgdorf, Germany) (1 mg/kg IV every 24 h), and amoxicillin and clavulanic acid (Amoxclav Hexal^®^, Hexal AG, Holzkirchen, Germany) (20 mg/kg every 12 h IV). On day 2, the condition of the dog deteriorated. The WBC count continued to rise; liver enzyme activities, hyperbilirubinemia, and renal values deteriorated; and a disseminated intravascular coagulation was suspected. The dog was euthanized due to a bad prognosis. A necropsy was performed.

**Figure 3:**
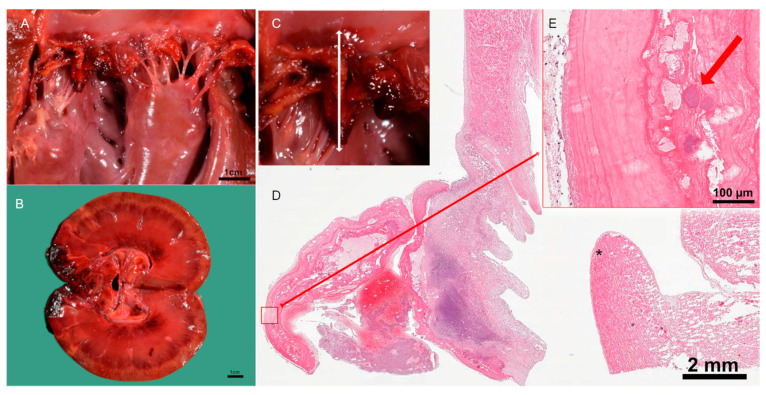
Pathological examination of the kidney and heart. (**A**) Post-mortem examination of mitral valve endocarditis. (**B**) Post-mortem examination of renal infarcts. (**C**) Location of the vegetation in macro image of the mitral valve. (**D**) Cross-section of the inflamed heart valve, * myocardium, and papillary muscle. (**E**) Intralesional coccoid bacteria with fibrin in pink and bacterial colonies in purple, indicated by red arrow.

The pathological examination identified a typical picture of sepsis and confirmed most of the clinical diagnoses. The main finding was severe thrombotic valvular endocarditis of the mitral valve with intralesional coccoid bacteria detectable by histology (Figure 3). Further septic manifestations were present in the brain (encephalitis), kidneys (nephritis), and the spleen (splenitis), each with purulent-necrotizing character and consistent with embolic pathogenesis. Infarcts in the kidneys and spleen occurred consecutively. An injury to the ear, which was suspected to be a bite injury, was a hypothetical portal of entry.

Blood cultures from two consecutive days were cultivated on Columbia blood agar (5% sheep blood). Examination of the plate after overnight incubation at 37 °C in aerobic conditions showed a pure culture of beta-hemolytic bacteria. No bacteria were isolated from the urine. Antibiotic susceptibility testing showed that the bacteria were susceptible to commonly used antibiotics such as β-lactams and had intermediate susceptibility to tetracyclines (Table 2). Identification of the bacteria was carried out via matrix-assisted laser desorption ionization/time of flight mass spectrometry (MALDI-TOF MS) coupled with Bruker Microflex LT and Flex Control (flexControl Version 3.4) as well as Biotyper (MBT Compass 4.1) software (Bruker Daltonics, Bremen, Germany). The bacteria were identified as *Streptococcus canis* with a score of 2.09. DNA was extracted from an overnight culture in BHI medium using the QIAamp DNA Mini Kit. Whole-genome sequencing (WGS) using Illumina MiSeq (Illumina Inc., San Diego, CA, USA) and subsequent assembly with assembleBAC was performed [1]. Multilocus sequence typing was performed via the PubMLST webtool, which revealed that this strain (denominated as IMT 49926) belongs to the ST35 type (Table 3).

Fluorescence in situ hybridization (FISH) of the heart valve tissue sections was performed to visualize the microbial biofilms in situ as described before [2,3]. The heart valve sample was briefly fixated, embedded in cold polymerizing resin, and sectioned. The sections were subjected to hybridization for two hours in a dark, humid chamber at 50 °C. The slides were rinsed with water, air-dried, and mounted for microscopy with an epifluorescence microscope (AxioImagerZ2; Carl Zeiss, Jena, Germany) equipped with narrow band filter sets (AHF-Analysentechnik, Tübingen, Germany). Sections were hybridized with the pan-bacterial, 16S rRNA-directed probe EUB338 (Cy3) [4] for visualization of most bacteria; the *Streptococcus* genus-specific FISH probes STREP1/2 (FITC) [5,6] for visualization of streptococci; and the nonsense probe (NON338) to exclude unspecific probe binding [7]. The nucleic acid stain DAPI (4′,6-diamidino-2-phenylindole) was applied as a counterstain to visualize the host cell nuclei and microorganisms that do not contain ribosomes anymore or contain too few ribosomes to be visualized by microscopy. Each hybridization experiment was controlled using bacterial positive reference strains and negative control strains with minimum mismatches in the probe target sequence.

**Figure 4:**
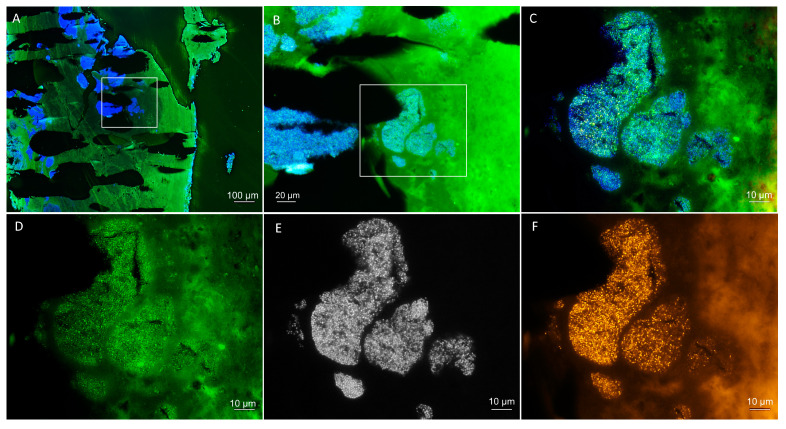
Fluorescence in situ hybridization (FISH) to visualize and identify the bacteria within the patient’s heart valve. (**A**) Overview of the heart valve showing extensive bacterial biofilms by DAPI staining in blue with the autofluorescent tissue background in green. (**B**) Magnification of a colonized region of the heart valve (marked in (**A**)) with a superimposition of the DAPI staining in blue and the *Streptococcus* genus-specific FISH probes in green (autofluorescent tissue background in green as well). (**C**) Further magnification of the colonized region (marked in (**B)**). (**D**–**F**) Identical field of view as (**C**), showing separate fluorescence filter sets for (**D**) *Streptococcus* genus-specific FISH probes in green, (**E**) DAPI DNA staining in black and white, and (**F**) bacteria with the pan-bacterial FISH probe EUB338 in orange. Nonsense probe not shown. Note the high signal intensity of the streptococci indicating a high ribosomal content, indicative of activity despite correct antibiotic therapy.

FISH showed extensive streptococcal biofilms present within the tissue (Figure 4). The strong FISH positive signal indicates a high ribosomal content and presumable bacterial activity despite antibiotic treatment.

## 2. Discussion

Infective endocarditis (IE) is a life-threatening infective disease that affects mostly middle-aged, larger male dogs [8]. The clinical signs of fever, lethargy, heart murmur, and weight loss are nonspecific and might be overlooked initially. Therefore, it is likely that the prevalence of IE is higher than reported. The dog described here had two out of five Okano SIRS criteria (rectal temperature > 39.7 °C, leukocytes > 12 × 10^9^/L) and fulfilled two major clinical criteria (new heart murmur, as no heart murmur was described before the referral to the internal medicine unit, and echocardiogram positive for IE) and two minor clinical criteria (one positive blood culture, fever) of the modified Duke criteria for diagnosis of IE. He additionally fulfilled the pathological criteria for definite infective endocarditis, as histology showed active endocarditis [9]. Measurement of the serum cardiac troponin-I concentration can be helpful to differentiate infective endocarditis from other differential diagnoses such as myxomatous mitral valve disease. Dogs with infective endocarditis have significantly higher troponin concentrations than dogs with mitral valve disease (stage B2) or dogs with immune-mediated diseases [10]. The authors concluded that a cut-off of >0.625 ng/mL is supportive for infective endocarditis. In the patient described here, an increase in cardiac troponin-I concentration is influenced by a decrease in renal function in addition to endomyocarditis. In humans, high levels of cardiac troponin-I are associated with a bad prognosis of IE, but this correlation is not shown for dogs [10,11].

The proposed mechanism for IE is predisposition to the formation of bacterial vegetations by a damaged and infected endothelium. The establishment of bacterial vegetation is a complex process that is at this time only partially understood. It is believed that an initial step in the pathogenesis of IE is the adherence of bacteria to damaged heart valve tissue. Platelets aggregate to the endocardial injury, followed by the accumulation of innate immune cells, tissue factors, and cytokines. This results in a vegetation, a form of mature biofilm, that consists of platelets, fibrin, thrombin, bacteria, other proteins, nutrients, and immune cells [12]. Oppegard and colleagues have shown that there is a positive correlation between the ability of *S. pyogenes*, a close relative of *S. canis*, to bind to the extracellular matrix protein fibronectin and the clinical expression of IE [13]. Whole genome sequencing and subsequent bioinformatic analyses of strain IMT 49926 accordingly confirmed the presence of genes encoding fibronectin-binding proteins. The ability to bind to serum proteins is also a characteristic of pyogenic streptococci. In previous studies of *S. canis*, we demonstrated the interaction of the bacteria with plasminogen or fibrinogen through SCM, the *S. canis* M protein; it thus participates directly in the process of intravascular thrombus formation [14,15]. As part of the thrombus, streptococci are protected from the attacks of the cellular immune system and are able to establish bacterial vegetations on the heart valves. No research has been performed on the duration of IE development in dogs. In human medicine, healthcare-associated infective endocarditis can develop within 48 h [16]. Thromboembolic complications as seen in this dog are common. The main affected organs are the lungs, kidneys, spleen, brain, and the distal part of the aorta [8,17,18,19].

According to the literature, blood cultures for the diagnosis of sepsis are positive in up to 46% of dogs with IE. The most common reason for a false negative blood culture is pretreatment with antibiotics. In this case, the blood culture was positive despite pretreatment with doxycycline (according to the antibiogram intermediate sensitive). The most common bacterial isolates from IE cases in dogs are *Staphylococcus* spp. and *Streptococcus* spp. [20]. *Streptococcus canis* (*S. canis*) was found to be involved in almost a quarter of endocardium infections and typically affected the mitral valve [21]. 

Bacteria can enter the bloodstream in many ways, such as through superficial infections, wounds, or through surgery. Streptococcal septicemia is often a sequel to localized infections in older dogs [22]. *Streptococcus* spp. colonize the skin, genital mucosae, and gastrointestinal tract in healthy dogs and can replicate quickly in the event of infection [22]. When the bacteria pass the skin barrier, they can use various surface proteins to interact with cells and form a biofilm [23]. In this case, an ear wound was the prime suspect of causing bacterial entry to the bloodstream, as there was no history of infections or surgery. However, an endogenous origin cannot be excluded. Pretreatment with glucocorticoids was likely a predisposing factor.

Using MALDI-TOF mass spectrometry, the causative bacteria could be identified to be *S. canis*. Multilocus sequence typing showed that this was an *S. canis* strain with sequence type 35. *S. canis* belongs to the Lancefield group G streptococci and is generally associated with both superficial and severe infection. It can form large colonies and is capable of β-hemolysis [22,24]. *S. canis* can colonize the skin and mucosae of asymptomatic individuals. One study found *S. canis* in 6.5% of healthy dogs (*n* = 35/359) and 5.9% of healthy cats (*n* = 10/169), mainly in the rectum, pharynx, and oral cavity [25].

Standard treatment for IE is the administration of antimicrobial drugs for at least eight weeks, preferably with intravenous administration in the first week of treatment. Empirical broad-spectrum antibiotic therapy is started while waiting for the results of blood cultures and continued in cases where the pathogen is not identified. In this case, amoxicillin/clavulanic acid was administered IV. The recommended antibiotic for acute *S. canis* IE cases is a high dose of ampicillin or ceftriaxone, and amoxicillin or amoxicillin/clavulanic acid in chronic cases. Ref. [20] Biofilm formation on the heart valve and the development of vegetations was in a more progressed, mature state, which was shown in the FISH images post-mortem and confirmed the IE diagnosis. These biofilms make antibiotic treatment less likely to be successful because biofilm-associated bacteria are recalcitrant to antibiotics [26]. Microorganisms in biofilms have a 10- to 1000-fold increased minimum inhibitory concentration compared with the same microorganisms growing planktonically [27]. Although the pathogen was susceptible to the used antibiotic in vitro (Table 2), it had intermediate susceptibility to the doxycycline that was used in the previous treatment. Antibiotic susceptibility testing in vitro does not account for biofilm formation. To account for higher MIC values in biofilms, antibiotic treatment in IE relies on high serum concentrations of antimicrobials [20]. Antibiotic tolerance in biofilms has different mechanisms, including failure of the antibiotic to penetrate biofilms, persister cells, altered metabolism, and the matrix itself [28].

Disruption of the biofilm could be a way to improve antibiotic treatment success in IE. Cyclic diguanylate (C-di-GMP) can interfere with bacterial gene regulation during biofilm formation and has been shown to reduce biofilm formation and adhesion to human epithelial cells in vitro in a study with *S. aureus* [29]. Another study showed that hyperbaric oxygen treatment could improve the outcome of antibiotic treatment [30]. We propose that treatment could be improved by optimizing antibiotic dosage combined with the use of antibiofilm agents. However, this strategy remains to be investigated in dogs with IE. Despite the susceptibility of this *S. canis* strain to amoxicillin and clavulanic acid, the condition of the dog worsened over the first 48 h and the dog was euthanized.

In human medicine, the gold standard treatment for IE is valve replacement surgery. In veterinary medicine, this surgical treatment is carried out rarely, and only in mitral valve regurgitation, which makes improving medicinal treatment and diagnosis for IE even more crucial [31]. Supportive treatment with antithrombotics is recommended but was contraindicated in this case due to severe thrombocytopenia.

The overall prognosis of dogs with IE is poor, with the prognosis being worse for dogs with an infected aortic valve compared with dogs with an infected mitral valve [20]. The most common cause of death in dogs with IE is thrombi, fragments of vegetations that enter the bloodstream. Treatment of IE consists of high-dose, long-term antibiotics and is successful in curing IE in less than half of the cases [8]. Within IE vegetations, microorganisms are present as biofilm-like aggregates and show various degrees of bacterial activity. Even after long-term antibiotic therapy, biofilms can be identified on surgically removed valves in human IE cases. Therefore, persistent cases of IE, even after initial infectious disease control, can be related to biofilm formation on the heart valve [32]. In a recent study with 113 dogs with IE, the survival to 1 month was 54% [8]. Negative prognostic factors in this study were the development of congestive heart failure, thromboembolic events, and acute kidney injury. In the patient described here, unfortunately, there were no blood and urine cultures examined at the beginning of clinical signs. An earlier diagnosis of sepsis and infective endocarditis might have influenced the treatment plan and the course of the disease. The dog received an immunosuppressive dosage of prednisolone and doxycycline, an antibiotic that was not optimal, as *S. canis* showed intermediate susceptibility to tetracyclines. Therefore, in patients with fever of uncertain origin, blood and urine cultures should be examined. The results should be available quickly to ensure the best possible treatment. Moreover, echocardiography should be part of the diagnostic work-up, especially when a new heart murmur emerges.

## 3. Conclusions

This case report shows the importance of early diagnosis and stresses the need for research on faster and more efficient medical treatment of IE caused by *S. canis*. We show that a combination of blood culture, MALDI-TOF MS, and MLST could determine the causative bacteria and its sequence type in 48–72 h to confirm the diagnosis of IE. This is a valuable tool for treatment because it is of high concern to use the right antimicrobial drugs. It is also of worth to be able to point out the risks of certain bacterial strains and investigate their characteristics for research purposes. In addition, antibiotic susceptibility testing is worthwhile for microorganisms with unknown susceptibility.

## Figures and Tables

**Table 1:** Clinicopathological findings in an 8-year-old Rhodesian Ridgeback with streptococcal sepsis.

Parameter	Day 1	Day 2	Reference Interval
Leukocytes (×10^9^/L)	**53.8**	**88.8**	5.6–14
Hematocrit (L/L)	0.44	**0.38**	0.42–0.56
Hemoglobin (g/L)	15.9	**12.5**	14.7–19.9
MCV (fL)	66	71	62–72
MCHC (g/dL)	35	33	32–36
Platelets (×10^9^/L)	**3**	**5**	165–400
Reticulocytes/µL	ND *	**139,800**	<60,000 non-reg.
Band neutrophils (×10^9^/L)	**2.15**	**1.77**	0.6
Segmented neutrophils (×10^9^/L)	**48.4**	**70.2**	3–11
Eosinophils (×10^9^/L)	0	0	−0.6
Lymphocytes (×10^9^/L)	1.07	1.77	1.0–4.0
Monocytes (×10^9^/L)	**2.2**	**15.1**	−0.5
Sodium (mmol/L)	142	150	140–150
Potassium (mmol/L)	4	3.7	3.6–4.8
Glucose (mmol/L)	**6.8**	**8.9**	4.5–6.2
Creatinine (µmol/L)	**221**	**288**	53–124
Urea (mmol/L)	**18**	**29**	3.5–10
Phosphorus (mmol/L)	**2.0**	**2.55**	0.96–1.6
Calcium (mmol/L)	2.5	2.5	2.5–2.9
ALT (U/L)	61	59	−76
AP (U/L)	**341**	**421**	−97
AST (U/L)	**43**	**158**	−41
Bilirubin (µmol/L)	**11**	**82.4**	−5.1
Protein (g/L)	**78**	60	54–66
Albumin (g/L)	**23**	**18**	28–36
DGGR lipase (U/L)	**3837**	**469**	−260
PT * (s)	24.4	**26.9**	16.5–25
aPTT * (s)	**21.9**	**23.5**	10–13.1
CRP (mg/L)	**149**	**143**	−10
Troponin I (ng/mL)	**3.45**	ND	−0.08

* aPTT: Activated thromboplastin time; ND: Not done; PT: Prothrombin time. Values outside the reference interval are indicated in bold.

**Table 2:** Antibiogram of *Streptococcus canis* strain.

Class	Antibiotic	Susceptibility ^#^
Β-lactams	Ampicillin	S
	Amoxicillin	S
	Amoxicillin/clavulanic acid	S
	Benzylpenicillin	S
	Cefalexin	S
	Cefazolin	S
Tetracyclines	Doxycycline	I
	Tetracycline	I
Macrolides	Azithromycin	S
	Clarithromycin	S
	Erythromycin	S
	Chloramphenicol	S
Other	Clindamycin	S
	Trimethoprim/Sulfamethoxazole	S

^#^ S: susceptible, I: intermediate.

**Table 3:** Multilocus sequence typing shows that this *S. canis* strain belongs to group 35.

Locus	Contig	Match	Allele
gki	Bac-Gen_S28_2_Contig_25	Exact	6
gtr	Bac-Gen_S28_2_Contig_25	Exact	1
murl	Bac-Gen_S28_2_Contig_2	Exact	1
mutS	Bac-Gen_S28_2_Contig_56	Exact	6
recP	Bac-Gen_S28_2_Contig_23	Exact	4
xpt	Bac-Gen_S28_2_Contig_14	Exact	10
ygiZ	Bac-Gen_S28_2_Contig_23	Exact	6
	MLST		35

## Data Availability

Publicly available datasets were analyzed in this study. This data can be found here: https://www.ncbi.nlm.nih.gov/bioproject/PRJNA945807 (accessed on 17 March 2023).
